# Ghrelin stimulates fatty acid oxidation and inhibits lipolysis in isolated muscle from male rats

**DOI:** 10.14814/phy2.14028

**Published:** 2019-04-08

**Authors:** Emily N. Kraft, Daniel T. Cervone, David J. Dyck

**Affiliations:** ^1^ Department of Human Health and Nutritional Sciences University of Guelph Guelph Ontario Canada

**Keywords:** Acetyl CoA carboxylase, ghrelin, hormone sensitive lipase, lipolysis, oxidation, skeletal muscle

## Abstract

Ghrelin is classically known as a central appetite‐stimulating hormone but has recently been recognized to have a significant role in peripheral tissue energy metabolism. However, the direct effects of ghrelin on skeletal muscle, a major site for glucose and lipid disposal, remain understudied. We found that the two major ghrelin isoforms, acylated and unacylated ghrelin, were able to significantly increase skeletal muscle fatty acid oxidation (~20%) while incorporation of fatty acids into major lipid pools remained unchanged. The increase in fatty acid oxidation was accompanied by increases in acetyl‐CoA carboxylase phosphorylation, a downstream target of AMPK. Ghrelin isoforms had no independent effect on lipolysis under unstimulated conditions, but nearly completely abolished epinephrine‐stimulated lipolysis. This effect was generally, but not consistently related to a blunting in the phosphorylation of HSL activation sites, Ser660 and 563. Taken together, these findings suggest that ghrelin isoforms have a direct, acute effect on fatty acid oxidation and lipolysis.

## Introduction

Ghrelin is classically known as a central appetite‐stimulating hormone, but may also have a significant role in peripheral tissue energy metabolism. There are two main ghrelin isoforms; unacylated (UnAG) and acylated ghrelin (AG). AG is generally thought to be the more bioactive isoform, although recent studies suggest an important role of UnAG in energy metabolism (Granata et al. 2012). It has been proposed that ghrelin may be important in maintaining blood glucose levels during times of energy restriction. This is supported by the fact that mice lacking ghrelin‐O‐acyltransferase (GOAT), the enzyme responsible for the synthesizing AG, are unable to maintain blood glucose during calorie restriction (Zhao et al. 2010). This effect is likely due, in part, to ghrelin's ability to increase growth hormone (GH) which in turn is thought to increase hepatic glucose output and adipose lipolysis, of which the latter may decrease muscle glucose uptake (Kim and Park 2017). However, with prolonged fasting, that is, during the last 24 h of a 61 h fast, AG levels decline (Liu et al. 2008), possibly due to a decreased availability of gut‐derived medium chain fatty acid for acylation. Furthermore, the entrained spikes in AG that would normally occur before mealtime also disappear (Liu et al. 2008). It has been postulated that ghrelin may act more as a nutrient sensor or cue (particularly for lipids) rather than as a hunger signal in the face of persistent fasting or food deprivation (Kirchner et al. 2009).

Given that ghrelin rapidly increases prior to entrained mealtimes, it is possible that ghrelin may act as part of a preparatory response to facilitate the clearance and metabolism of ingested glucose and lipids. Skeletal muscle is a major site for glucose and lipid disposal; however, the direct effects of ghrelin on this tissue remain understudied. Studies utilizing in vivo AG and UnAG injections in male rats over several days have generally shown improved muscle glucose handling with concomitant increases in Akt phosphorylation (Barazzoni et al. 2007; Tam et al. 2015; Cappellari et al. 2016), as well as GLUT4 mRNA (Cappellari et al. 2016) and protein (Tam et al. 2015). In male humans, the acute administration of AG decreases both basal and insulin‐stimulated whole‐body glucose clearance (Vestergaard et al. 2008). However, conclusions from in vivo studies are potentially confounded by secondary effects on GH. Other studies have provided evidence for a direct, acute stimulatory effect of AG or UnAG on glucose uptake in various muscle cell models, including murine C2C12 cells and rat myoblasts (Gershon and Vale 2014; Han et al. 2015; Cappellari et al. 2016). The myoblasts utilized in these studies (Granata et al. 2012; Cappellari et al. 2016) originated from male rats. In our own laboratory, we were unable to demonstrate any direct effect of either AG or UnAG on glucose uptake in isolated, mature skeletal muscle derived from male rats regardless of the presence or absence or insulin, or muscle fiber type (Cervone and Dyck 2017). Ghrelin's effects on muscle metabolism may be attributable to its interaction with the Corticotropin‐Releasing Factor (CRF) receptor, CFR‐R2 (Gershon and Vale 2014) as demonstrated in muscle C2C12 cells.

The effects of ghrelin on skeletal muscle fatty acid (FA) metabolism are less well studied. Daily peripheral AG injections in male mice appears to reduce whole‐body fat utilization, as evidenced by a greater respiratory quotient (Tschop et al. 2000). However, other studies have shown a reduction in triacylglycerol (TAG) content in muscle suggesting greater lipolysis (and potentially utilization) following 4 days of AG injections in male rats (Barazzoni et al. 2005), as well as in isolated myoblasts derived from male rats incubated with AG for 12 h (Han et al. 2015). The direct effects on muscle lipolysis and its regulation have not been examined, although several studies have now identified that ghrelin can inhibit isoproterenol‐stimulated lipolysis in rat adipocytes from male rats (Muccioli et al. 2004; Baragli et al. 2011) and adipose tissue organ culture derived from male rats (Cervone et al. 2018).

The objective of this study was to determine the direct effects of ghrelin on FA metabolism (oxidation, incorporation into lipids, lipolysis) in isolated, mature skeletal muscle obtained from male rats. Furthermore, we sought to gain insight as to whether these effects were dependent on glucose availability, which can change significantly after meal consumption, and potentially alter FA utilization. Finally, we examined the potential effect of ghrelin on FA metabolism in muscles of different oxidative potential (soleus, slow oxidative; extensor digitorum longus, fast glycolytic).

## Methods

### Materials and reagents

Reagents, molecular weight markers, and nitrocellulose membranes for SDS‐PAGE were purchased from Bio‐Rad (Mississauga, ON, Canada). Western Lightning Plus enhanced chemiluminescence (ECL) was purchased from Perkin Elmer (NEL105001EA). The following primary antibodies were purchased from Cell Signalling Technology: phospho‐HSL (Ser563, catalog no. 4139; Ser660, catalog no. 4126; Ser565, catalog no. 4137), total HSL (catalog no. 4107), phospho‐ACC (Ser79 catalog no. 3661), and total ACC (catalog no. 3662). Horseradish peroxidase‐conjugated donkey anti‐rabbit antibody was obtained from Jackson ImmunoResearch Laboratories (West Grove, PA, catalog no. 711‐035‐152). Free glycerol was measured using a commercially available fluorometric kit from BioVision (catalog no. K630–100). Medium 199 (catalog no. M3769), benzethonium hydroxide solution (catalog no. B2156), PMSF (catalog no. 78830), protease inhibitor cocktail (catalog nos. 78830 and 9599), and epinephrine hydrochloride (catalog no. E4642) were purchased from Sigma‐Aldrich Canada Co. Samples were homogenized in a Qiagen TissueLyser LT (cat. No 85600). AICAR was purchased from Cayman Chemical Co. (catalog no. 10010241‐100). Palmitic acid [1‐14C] was purchased from American Radiolabelled Chemicals (product no. 0172A). Acylated (catalog no. H‐4862) and unacylated (catalog no. H‐6264) rat ghrelin were sourced from Bachem (Torrance, CA).

### Animals

All procedures were carried out in accordance with the guidelines of the Canadian Council of Animal Care and were approved by the Animal Care Committee at the University of Guelph. Animals were anesthetized with sodium pentobarbital (6 mg/100 g body weight) prior to surgical interventions and all efforts were made to prevent discomfort. Male Sprague‐Dawley rats (200–250 g, approximately 7 weeks in age) were purchased from Charles River Laboratories. Animals were housed 2 to 4 per cage and kept in a 22 to 24°C environment. Animals were on a 12:12‐h reverse light‐dark cycle and fed standard rodent chow (Teklad Laboratory Diet, Envigo) ad libitum. Animals were given a minimum of 1 week to acclimate to their environment prior to any procedures.

### In vitro experiments for determination of FA oxidation and incorporation, lipolysis and signaling

On experiment days, after an overnight fast, animals were provided with food at 6:00 am which marked the beginning of their active phase. Animals were given ~1 h to feed, and a reduction in the volume of food was visually confirmed. Food was removed at 7 am, approximately 2 h prior to anaesthetization to allow insulin levels to return to normal concentrations, which we have previously confirmed (Cervone et al. 2018). This two hour period also significantly decreased circulating ghrelin compared to the overnight fasted condition (pre‐feeding, 0.92 ± 0.14 ng/mL vs. post‐feeding, 0.36 ± 0.03 ng/mL; *P* < 0.05), thereby eliminating exposure to an already elevated ghrelin as a confounding experimental factor. Experimental procedures began at 9 am. Animals were anaesthetized with an intraperitoneal injection of sodium pentobarbital. Soleus (SOL, oxidative) and extensor digitorum longus (EDL, glycolytic) muscles from hindlimbs were stripped lengthwise and excised with tendons intact, and then weighed. Muscles were then equilibrated for 30 min at 30°C in oxygenated (95% O_2_–5% CO_2_) medium 199 modified with 1 mmol/L palmitate and 4% fatty acid free bovine serum albumin in all experiments except those used exclusively for signaling/Western blotting. For these experiments, 1% fatty acid free bovine serum albumin was used in order to prevent the development of a gross non‐specific band around 50‐60 kDa.

#### Fatty acid oxidation

Muscles were incubated with the addition of 0.5 *μ*Ci/mL [1‐14C] palmitic acid, and either 5 mmol/L or 10 mmol/L glucose. Since glucose availability can potentially affect FA oxidation, glucose concentrations representing a normal (premeal) and higher (postmeal) value were used. Four muscle strips from each animal were obtained and randomly assigned to each of the following groups: (1) control with no additional treatment, (2) AICAR (positive control, 2 mmol/L), (3) acylated ghrelin (AG, 150 ng/mL) or (4) unacylated ghrelin (UnAG, 150 ng/mL) for a 1 h incubation. Previous work from our laboratory has confirmed the stability of both AG and UnAG in incubation media for at least 2 h [96]. The concentration of ghrelin chosen is supraphysiological but has been shown previously to elicit significant metabolic effects in isolated tissues, including CaMKII phosphorylation in muscle (Cervone and Dyck 2017) and an antilipolytic effect in adipose tissue (Cervone et al. 2018). Work by others examining the metabolic effects of ghrelin in isolated C2C12 muscle cells have utilized a dose of 100 nmol/L, or 337 ng/mL (Gershon and Vale 2014). Muscles strips were incubated in glass vials for 1 h. Following the incubation, 2 mL of 1M sulfuric acid was added via syringe directly to the medium and muscle, and the released ^14^CO_2_ gas was trapped in an Eppendorf tube containing benzethonium hydroxide over a 2‐h period. Eppendorf tubes containing the benzethonium hydroxide were counted via standard liquid scintillation techniques. Tendons were cut from the muscle and their weight was subtracted from the initial muscle weight obtained prior to incubation.

#### Fatty acid incorporation into phospholipids, diacylglycerol and triacylglycerol

Muscles were incubated under identical conditions as for the determination of FA oxidation, except that no radiolabelled palmitate was included. Following incubations, muscles were placed in 5 mL of 2:1 chloroform:methanol solution and homogenized. The homogenate was centrifuged at 10,000 *g* for 10 min (4(C). The supernatant was removed, to which 2 mL of ddH2O was added, shaken gently for 10 min and re‐centrifuged, and the bottom layer of chloroform was removed. One milliliter of pure chloroform was added to the centrifuge tube to dissolve any remaining lipids. This was then added to the initially isolated chloroform. Samples were warmed at 30(C and evaporated under a stream of nitrogen gas. Once samples were completely evaporated, 100 *μ*L of 2:1 chloroform:methanol solution was added to the dissolved lipids for spotting. Unlabelled phosphotidyl choline, dipalmitin, and tripalmitin was added to the chloroform:methanol solution to facilitate band detection. Fifty microliters of dissolved lipids were spotted on a thin layer chromatography plate which was developed for 45 min in heptane:isopropylether:acetic acid (60:40:3). The resolved plates were allowed to completely dry and lightly sprayed with chlorofluorescein dye (0.02% w/v in ethanol) to visualize the lipid bands. The individual bands were scraped off into scintillation vials and counted.

#### Lipolysis and glycerol quantification

Only soleus muscle was used for the assessment of lipolysis as previous work in our laboratory has shown that EDL does not respond significantly to epinephrine (Peters et al. 1998). Incubation conditions included (1) control, (2) epinephrine (Epi, 1 *μ*mol/L), (3) Epi + AG (1 *μ*mol/L, 150 ng/mL), and Epi + UnAG (1 *μ*mol/L, 150 ng/mL). Glycerol concentration in the incubation media was used as an index of lipolysis and was measured fluorometrically in triplicate.

#### Signalling experiments and western blotting

Pre‐incubations were 30 min for all signaling experiments. Incubations for ACC signaling were 30 min in order to capture the maximal phosphorylation of this enzyme. Incubations to assess HSL phosphorylation were for one hour to match the duration used to assess lipolysis. Muscles were blotted, frozen in liquid nitrogen, and stored at −80°C until analyses were performed. For western blot analyses, muscles were chipped into 20 to 30 mg pieces under liquid nitrogen and homogenized in a 500 uL of ice‐cold cell lysis buffer supplemented with PMSF and protease inhibitor cocktail. Samples were homogenized in a Qiagen TissueLyser LT for three, 3‐min intervals and then centrifuged at 1500 *g* for 15 min. Protein concentration of the supernatant was determined using the bicinchoninic acid method and equal amounts of protein were separated on 10% gels to assess the protein content of p‐HSL Ser660, p‐HSL Ser563, p‐HSL Ser565, total HSL, and 5% gels for p‐ACC Ser79 and total ACC. Proteins were transferred to nitrocellulose membranes at a constant 200 mA per tank and subsequently blocked in Tris‐buffered saline‐0.01% Tween (TBST) supplemented with 5% nonfat dry milk for 1 h at room temperature with gentle shaking. Membranes were then washed and incubated at 4°C overnight in primary antibodies diluted 1:1000 in TBST with 5% BSA. The following day, blots were washed with TBST and then incubated in TBST‐1% nonfat dry milk supplemented with 1:2000 horseradish peroxidase conjugated donkey ant‐rabbit secondary antibody for 1 h at room temperature. Bands were visualized using ECL and quantified using Alpha Innotech software. Western blots were normalized against vinculin to account for any irregularities in protein loading. Phosphorylated proteins were also normalized to their respective total protein.

### Statistical analysis

A repeated measure one‐way ANOVA and Fisher's LSD was used for analysis of FA oxidation and FA deposition. A two‐way repeated measure ANOVA and Fisher's LSD was used to analyze the free glycerol assay in order to examine the interaction of ghrelin and epinephrine independently and in combination. A one‐way repeated measure ANOVA and Fisher's LSD was used for analysis of Western blotting. Statistical analysis was accepted at *P* < 0.05.

## Results

### Ghrelin directly stimulates fatty acid oxidation in oxidative and glycolytic muscle

**Figure 1:**
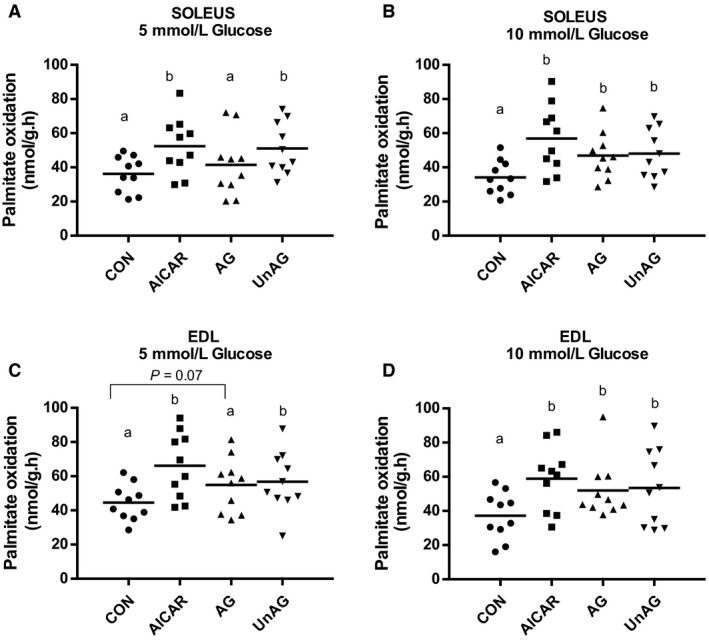
Ghrelin stimulates fatty acid oxidation. Labeled palmitate oxidation in soleus and EDL at 5 mM (A and B) and 10 mM glucose (C and D). Individual data points are shown with the mean indicated. *n* = 10 per group. Data were considered significant at *P* ≤ 0.05. Groups not sharing a letter are statistically different from each other.

AICAR, which served as a positive control for the assessment of FA oxidation, stimulated palmitate oxidation in both soleus and EDL (Fig. 1) regardless of the glucose concentration (*P* < 0.01). At 5 mmol/L glucose, UnAG stimulated oxidation in both soleus (*P* < 0.01) and EDL (*P* < 0.03). AG also tended to stimulate palmitate oxidation in EDL (*P* = 0.07). At 10 mmol/L glucose, both ghrelin isoforms significantly stimulated palmitate oxidation in soleus (*P* < 0.01) and EDL (*P* < 0.05).

### Ghrelin does not alter fatty acid incorporation into triacylglycerol, diacylglycerol, or phospholipids

**Figure 2:**
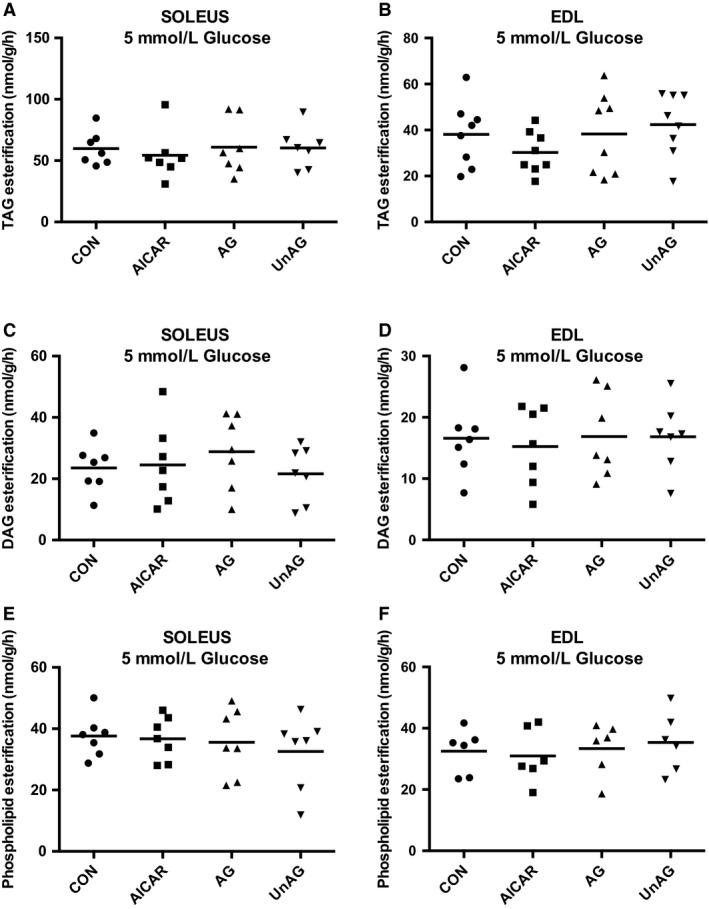
Ghrelin does not alter palmitate incorporation into TAG, DAG, or phospholipids. Labeled palmitate incorporation into triacylglycerol (TAG), diacylglycerol (DAG) or phospholipids (PHOS) in soleus (A, C, E) and EDL (B, D, F) with 5 mmol/L glucose. Similar results were observed at high glucose concentration (10 mmol/L) (Table 1). Individual data points are shown with the mean indicated. *n* = 7–8 per group. Data were considered significant at *P* ≤ 0.05.

No significant differences in the incorporation of labelled palmitate into triacylglycerol (TAG), diacylglycerol (DAG), or phospholipids (PL) were observed with any treatment in either the 5 or 10 mmol/L glucose environments in soleus or EDL. Fatty acid incorporation into the major lipid pools is shown Figure 2 for the 5 mmol/L glucose condition. Results were similar at 10 mmol/L glucose and shown in Table 1.

**Table 1:** ^14^C labeled palmitate incorporation into major lipid pools in SOL and EDL at 10 mM glucose buffer concentration

	SOL				EDL			
	CON	AICAR	AG	UnAG	CON	AICAR	AG	UnAG
TAG (nmol/g/hr)	52.4 ± 3.5^a^	55.1 ± 6.0^a^	71.0 ± 6.2^a^	63.1 ± 7.3^a^	46.5 ± 6.1^a^	41.7 ± 6.7^a^	52.2 ± 8.3^a^	57.9 ± 8.0^a^
DAG (nmol/g/hr)	23.8 ± 5.0^b^	20.9 ± 2.9^b^	25.8 ± 6.4^b^	20.8 ± 4.5^b^	21.7 ± 3.0^b^	19.2 ± 2.7^b^	28.3 ± 5.5^b^	23.7 ± 3.0^b^
PHOS (nmol/g/hr)	37.1 ± 4.8^c^	30.9 ± 2.2^c^	35.3 ± 1.7^c^	44.2 ± 7.3^c^	35.3 ± 3.4^c^	38.9 ± 5.6^c^	40.2 ± 4.8^c^	50.2 ± 6.5^c^

Data are expressed as the mean ± SE. *n* = 7 per group. Data was considered significant if *P* < 0.05. Groups not sharing a letter are considered statistically different from each other. SOL, soleus; EDL, extensor digitorum longus; TAG, triacylglycerol; DAG, diacylglycerol; PHOS, phospholipids; AG, acylated ghrelin; UnAG, unacylated ghrelin; SOL, soleus; EDL, extensor digitorum longus.

### Ghrelin blunts epinephrine‐induced lipolysis in oxidative muscle

**Figure 3:**
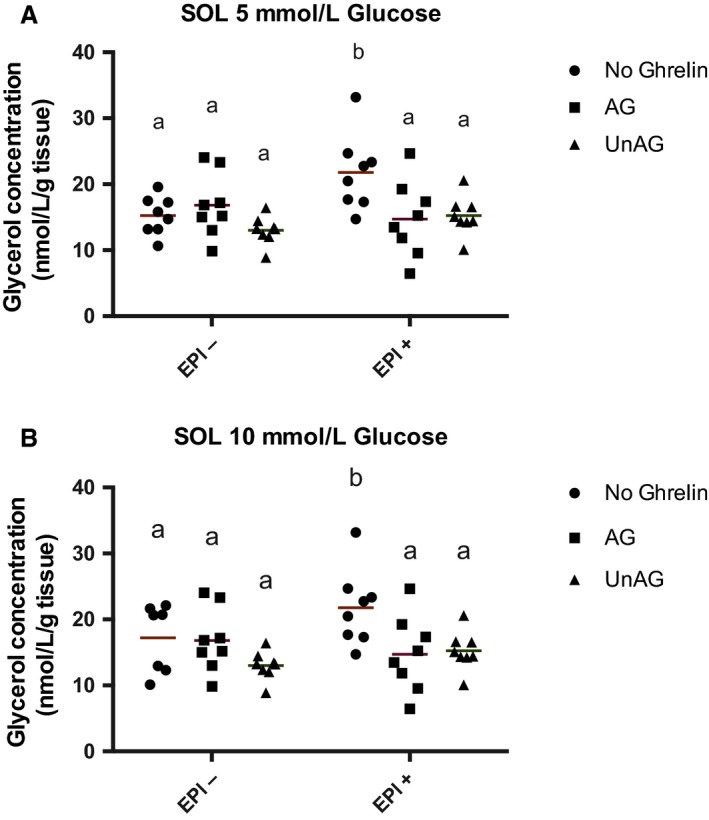
Ghrelin inhibits skeletal muscle lipolysis. Ghrelin alone does not stimulate glycerol release and blunts epinephrine stimulated lipolysis at normal (A) and high (B) glucose concentrations. Individual data points are shown with the mean indicated *n* = 7–8 per group. Data were considered significant at *P* ≤ 0.05. Groups not sharing a letter are statistically different from each other. Epi, epinephrine.

Previous work from our laboratory has demonstrated that epinephrine is able to significantly stimulate lipolysis in the soleus but not the EDL muscle [119]. Therefore, we examined only the soleus muscle to determine the role of ghrelin as a regulator of lipolysis. Epinephrine significantly increased glycerol concentration in the medium, an indicator of lipolysis, at both 5 mmol/L (*P* = 0.0002) and 10 mmol/L (*P* = 0.0016) glucose concentrations, compared to control (Fig. 3). Neither ghrelin isoform (AG, UnAG) was able to independently alter glycerol release compared to control, that is, in the absence of epinephrine. However, when either ghrelin isoform was added in the presence of epinephrine, a significant blunting of epinephrine's stimulatory effect on glycerol release was observed (*P* < 0.05).

### Ghrelin increases ACC phosphorylation in oxidative and glycolytic muscle

**Figure 4:**
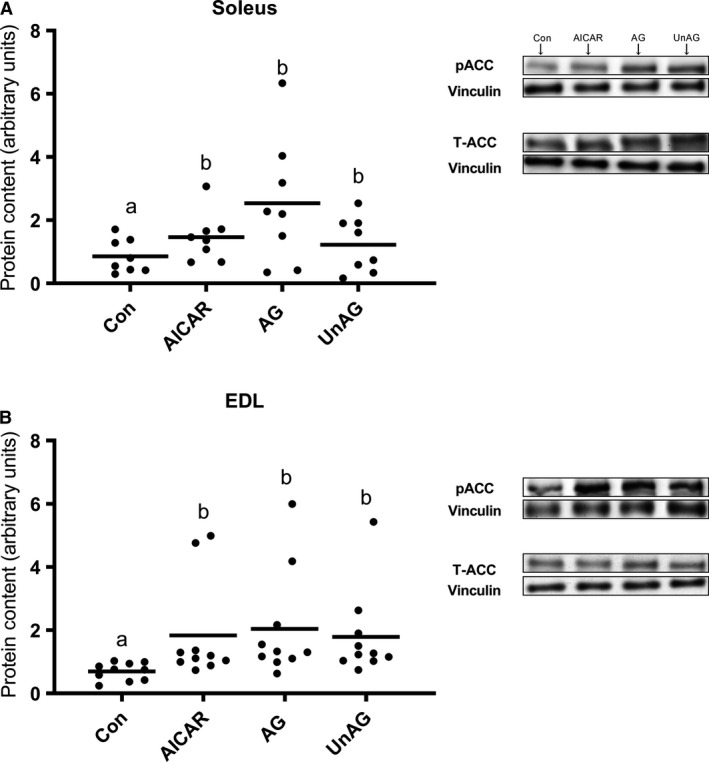
Direct effect of AICAR (AMPK agonist), AG and UnAG on the activation of the downstream AMPK target ACC in (A) soleus and (B) EDL skeletal muscle. Individual data points are shown with the mean indicated, in arbitrary protein units (phospho/total) normalized to vinculin. *n* = 8–10 per group. Data sharing a letter are not statistically different from each other. A value of *P* < 0.05 was considered statistically significant. AG, acylated ghrelin; UnAG, unacylated ghrelin; AICAR, 5‐aminoimidazole‐4‐carboxamide ribonucleotide; AMPK, AMP‐activated protein kinase; ACC, acetyl CoA carboxylase.

ACC phosphorylation was measured to assess activation of the AMPK axis. ACC phosphorylation is better maintained and less transient than AMPK phosphorylation, and considered to be an appropriate readout of activation of this axis. Since ghrelin was able to significantly stimulate FA oxidation regardless of the available glucose concentration, ACC signalling was assessed only at 5 mmol/L glucose. Both isoforms of ghrelin consistently increased ACC phosphorylation (*P* < 0.05), regardless of muscle fiber type (Fig. 4), which is consistent with the observed increase in FA oxidation.

### Ghrelin tends to blunt epinephrine‐stimulated HSL phosphorylaton in oxidative muscle

**Figure 5:**
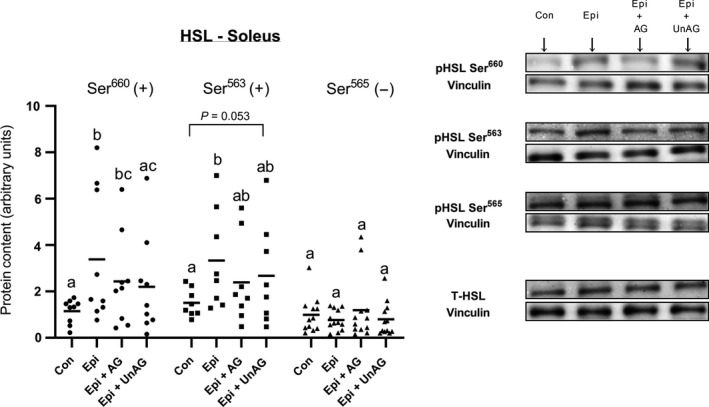
Direct effect of epinephrine alone or in combination with AG or UnAG on the phosphorylation of activating (Ser660/563) or inhibitory (Ser565) residues of HSL in oxidative, soleus skeletal muscle. Individual data points are shown with the mean indicated, in arbitrary protein units (phospho/total) normalized to vinculin. *n* = 8–12 per group. Data sharing a letter are not statistically different from each other. A value of *P* < 0.05 was considered statistically significant. HSL, hormone sensitive lipase; AG, acylated ghrelin; UnAG, unacylated ghrelin.

Epinephrine significantly (*P* < 0.005) increased the phosphorylation of both stimulatory residues (Ser660 and 563) on HSL (Fig. 5). This effect was diminished ~20 to 30% by the presence of ghrelin, although this was variable and statistically not significant. Consistent with a blunting effect of lipolysis, phosphorylation of Ser563 was no longer different from control when either AG or UnAG were combined with Epi. Similarly, Ser563 phosphorylation was no longer different from control when AG was combined with Epi. However, Ser660 phosphorylation remained significantly elevated from control when AG was combined with Epi. Furthermore, for the most part, phosphorylation of Ser563 and 660 were not significantly decreased from the Epi alone condition when AG and UnAG were added, somewhat complicating the functional interpretation. There was no significant effect of ghrelin on Ser565, an inhibitory phosphorylation site.

## Discussion

The direct effects of ghrelin on FA metabolism in skeletal muscle remain essentially unknown. Therefore, the purpose of this investigation was to determine ghrelin's direct effects on FA metabolism, including oxidation, incorporation into lipids, and lipolysis in isolated mature skeletal muscle. In this study, we utilized isolated skeletal muscle derived from male rats to assess changes in FA metabolism caused directly by acylated (AG) and unacylated (UnAG) ghrelin isoforms. This allowed us to directly examine the effects of ghrelin isoforms without the secondary effects or interaction of other hormones, such as GH, that normally occur in vivo. Muscle strips were incubated with either normal (5 mmol/L) or high glucose (10 mmol/L) concentrations in order to determine whether any potential effects of ghrelin on muscle FA metabolism might depend on glucose concentration, which can significantly change from the pre to post meal state. Here, we show that both ghrelin isoforms can directly stimulate palmitate oxidation and inhibit epinephrine‐induced lipolysis ex vivo, regardless of glucose concentration. The increase in palmitate oxidation in both muscle types was associated with an increase in the phosphorylation of ACC, a commonly used readout of AMPK activity. The blunting of epinephrine‐stimulated lipolysis by ghrelin generally coincided with a loss of phosphorylation of HSL on the activation residues serine 660 and 563, although this was not entirely consistent. These findings suggest that ghrelin may act to cause the muscle to preferentially shift to fat utilization, thereby either sparing glucose for other tissues, or in the context of a meal to promote the storage and maintenance of carbohydrate stores which are much smaller than adipose fat stores.

### Both ghrelin isoforms stimulate fatty acid oxidation in skeletal muscle

Previous work from our laboratory has demonstrated that neither ghrelin isoform has any effect on glucose uptake in isolated skeletal muscle from male rats (Cervone and Dyck 2017), suggesting that ghrelin's effect may be selective to FA metabolism. However, in fairness, glucose oxidation was not assessed in our previous study and we cannot confirm with certainty that this is not altered with ghrelin. To the best of our knowledge, we are the first to show that ghrelin directly and independently stimulates FA oxidation in mature skeletal muscle. This increase in FA oxidation ranged from approximately 15 to 42%. Interestingly, neither the oxidative capacity of the muscle, nor the availability of exogenous glucose appeared to affect this outcome.

The mechanism by which ghrelin acutely increases FA oxidation in skeletal muscle is unknown. We hypothesized that AG and UnAG would stimulate FA oxidation in skeletal muscle by phosphorylating AMPK and its downstream effector, ACC, which in turn relieves malonyl CoA inhibition on mitochondrial CPT1. In the current study, and consistent with our hypothesis, both ghrelin isoforms caused a significant increase in ACC phosphorylation, regardless of fiber type or glucose availability. This is consistent with our observation of increased FA oxidation. We chose to assess only ACC phosphorylation, and not AMPK, as AMPK phosphorylation in skeletal muscle is transient. ACC phosphorylation is better sustained and generally considered to be a robust indicator of AMPK axis activation. It should be noted that in a previous investigation from our laboratory, also using male rats, we did not observe a ghrelin‐induced increased in AMPK phosphorylation (Cervone and Dyck 2017) in identical muscle types. However, phosphorylation of the downstream target ACC was not assessed in that study. Therefore, whether ghrelin is able to stimulate AMPK albeit very transiently, or phosphorylates ACC through a different mechanism is not known. The inability to directly test the involvement of AMPK represents a limitation to the current study. Compound C has been used to inhibit AMPK, but has other effects on the cell including altering mitochondrial metabolism. The use of an AMPK muscle‐specific knockout model would be helpful, but this would require moving to a mouse model, which is beyond the scope of the current study. This could be a focus of future studies.

### Ghrelin does not affect fatty acid incorporation into lipid pools, but blunts adrenergic‐stimulated lipolysis in skeletal muscle

Previous studies have demonstrated that chronic AG injections for 4 days in male rats can reduce intramuscular TG content in mixed gastrocnemius (Barazzoni et al. 2005). This suggests the possibility that ghrelin may alter the rate of turnover of muscle lipid pools. In the current study, the net incorporation of FA into various muscle lipids was assessed. Surprisingly, neither ghrelin isoform significantly altered radiolabeled ^14^C palmitate incorporation into TAG, DAG or PL pools. However, one limitation of this technique is that only the net accumulation of ^14^C palmitate within a given lipid pool is quantified. Therefore, it is possible that the tracer is taken up and released in a more active sub‐pool, or that any release of labeled palmitate may end up in a different lipid pool. However, within the inherent limitations of the tracer technique, we were unable to demonstrate any net effect of ghrelin on FA incorporation into the major lipid pools of the muscle.

Previous work has shown that both AG and UnAG inhibit isoproterenol‐induced lipolysis in isolated male rat adipocytes (Muccioli et al. 2004), which is similar to what we observed in skeletal muscle in this study. Neither AG nor UnAG had any effect on basal lipolysis, but both isoforms blunted epinephrine‐induced lipolysis in the soleus muscle. This blunting was evident regardless of the glucose concentration. Hormone sensitive lipase (HSL) is a key lipolytic enzyme that is regulated by the reversible phosphorylation of various serine sites, including an inhibitory Ser565 residue. HSL can be inhibited by AMPK through the phosphorylation of Ser565. Epinephrine, via protein kinase A (PKA), phosphorylates stimulatory serine residues, 660 and 563 (Watt et al. 2006). Previous studies have shown that epinephrine is able to directly stimulate skeletal muscle lipolysis in oxidative muscle (Peters et al. 1998; MacDonald et al. 2013). In the current study, phosphorylation of Ser563 and 660 were increased with epinephrine treatment as expected. However, while there was a general 20–30% reduction in the phosphorylation of these sites when ghrelin was combined with epinephrine, this was not always statistically significant. Therefore, it is unclear whether the blunting of epinephrine‐stimulated lipolysis by ghrelin is due to a reduction in HSL phosphorylation. It should be noted, however, that changes in HSL phosphorylation in skeletal muscle, for example in humans in response to epinephrine infusion or exercise, are generally quite modest, that is, ~25% (Watt et al. 2003). It is also possible that ghrelin may be interacting with adipose tissue glycerol lipase (ATGL), which is the rate limiting step in triacylglycerol lipolysis. However, from our own experience, the quality of western blots using commercially available antibodies for ATGL phosphorylation sites is relatively poor making it difficult to detect small differences. Furthermore, whether phosphorylation of ATGL reflects actual lipolytic activity in muscle is debatable (Mason et al. 2012).

Taken together, the findings from this study may suggest that in isolated rat skeletal muscle, ghrelin isoforms directly stimulate FA transport/uptake (although this remains to be directly assessed), as seen with increases in ^14^C palmitate oxidation, and no changes in FA incorporation into lipids. Furthermore, ghrelin does not independently alter lipolysis. Some additional sites of FA regulation that future studies should look to examine include the sarcolemmal FAT/CD36 FA transporter, and mitochondrial CPT1. Intriguingly, the central administration of ghrelin has been shown to alter both FATCD/36 and CPT1 expression in the brain as well as in adipose tissue (Theander‐Carrillo et al. 2006; Velasco et al. 2017).

### Limitations and considerations

In the current study, incubation medium glucose was controlled at two concentrations, as glucose rapidly changes with food (carbohydrate) consumption and can potentially alter the balance of substrate utilization by muscle. As such, we felt it important to verify ghrelin's direct effect on muscle FA metabolism when glucose availability was altered. Of course, it can also be argued that numerous other important hormonal changes occur after nutrient consumption, primarily insulin, as well as others including incretins, etc. Circulating concentrations of lipids and amino acids would also markedly change depending on the macronutrient composition of the meal. Clearly, it was beyond the scope of this study to manipulate all of these potential factors. Nonetheless, we recognize that there are important factors that could dictate ghrelin's overall effects on muscle substrate utilization. Another limitation to this study is the use of only male rats. Indeed, the literature to date indicate a clear use of males (whether human, rat, mouse or derived cells) in the area of ghrelin's peripheral metabolic effects. Circulating ghrelin levels are reported to be greater in females (Makovey et al. 2007; Abu‐Farha et al. 2014). However, to the best of our knowledge, sex differences in muscle response to ghrelin has not been examined. Finally, the response of skeletal muscle to numerous hormones, including insulin, leptin, adiponectin and others is altered in the obese condition, typically as an impairment (Steinberg and Dyck 2000; Steinberg et al. 2002; Bruce et al. 2005). To the best of our knowledge, no work has yet examined whether this might also be the case with the response of skeletal muscle to ghrelin.

Although we have approached our research question from the perspective of ghrelin being a potential preparatory signal in advance of an increase in circulating nutrients at mealtime, it is also worthwhile to consider a potential role in the obese condition. Obesity is often referred to as an inflammatory state. Specific inflammatory compounds, such as TNFalpha, are known to stimulate adipose lipolysis (Green et al. 2004) potentially leading to elevated circulating fatty acids. We have also demonstrated that TNFα directly stimulates fatty acid deposition into the reactive diacylglycerol pool in isolated rat muscle (Bruce and Dyck 2004). Given our findings that ghrelin can blunt stimulated lipolysis, both in muscle and adipose tissue, and increase fatty acid oxidation in muscle suggests a potential beneficial effect. Indeed, there is one report that we are aware of demonstrating that acylated ghrelin can prevent high palmitate‐induced impairment in glucose uptake in myoblasts derived from male rats (Han et al. 2015). Whether ghrelin can antagonize the actions of TNFalpha in muscle should be investigated, as well as the potential role of ghrelin in protecting muscle from FA overload.

## Conflict of Interest

The authors have no conflict of interest to declare.
